# Premium on Babies

**Published:** 1880-06

**Authors:** 


### PREMIUM ON BABIES

*To the Committees of Agricultural Associations.*

GENTLEMEN,—It is our good fortune to live in an age and country characterized by invention and improvement in the useful arts and sciences. Valuable premiums are annually awarded to those persons who have devised the best implements of husbandry, for developing the capacity of the soil for useful production. Companies have been formed and agents sent abroad to import into our country the best horses, cattle, &c.

As man stands at the head of the animal creation, and as he is equally with the inferior animals, subject to influences, which improve or deteriorate ; to know what circumstances, regimen, habits, &c., are best calculated to improve man physically, becomes a matter of great importance. I say to improve man physically, because there is a connexion so close, a sympathy so mutual, existing between the mind and body, that the capacity of the former for exertion, depends in a great degree upon the healthy condition of the latter, and hence if we would regard man as an intellectual being only, we perceive the importance of understanding those laws of the animal economy, by an observance of which, physical power is developed and health preserved.

If these laws were generally understood and observed by the people of our country, the complaints of dyspepsia and nervous diseases, now so common, would be comparatively unknown; and the languid victims of disease, incapacitated for usefulness and enjoyment, would be far better enabled to fulfil the designs of Nature in their creation.

But how can the mass of the people be informed in regard to these important matters? I answer that you have it in your power, to direct the minds of the people in this portion of our country, to the improvement of the human race.

You award premiums to those, who exhibit at our annual fairs, the best horses, cows, and sheep: award premiums also to those parents, who will exhibit the finest children of a certain age. Understand me: by finest children, I do not mean the most intellectual or beautiful. The intrinsic difficulty of making such decisions, as well as impolicy of the thing, must be apparent to all. I do not mean the fattest, nor yet the largest, except so far as size is combined with the best form, to produce strength, action, and capacity for endurance. In regard to forming a correct judgment of the physical superiority of children, I apprehend that good judges of cattle, might not be good judges of children. Although it is not my purpose or province to furnish rules, by which children may be physically estimated, I will remark, that human beings should not have such an accumulation of a lipous tissue, as to interfere with the movement and strength of the individual. The muscles should not be too soft and yielding, but rather firm and resisting. The chest should be well developed, the lungs sufficiently capacious to aërate or arterialize the entire amount of venous blood in the system, in due time. The form and structure should be such, as before remarked, to combine strength with agility. The various parts of the system should be in due and symmetrical proportion, the best fitted to resist disease, and the vital organs so constructed and sustained as to perform their functions easily and efficiently. The unobstructed and perfect performance of these functions, constitutes health; while a failure of these organs to perform their functions, constitutes disease.

What a delectable sight it would be to see mothers, with the conscious pride of useful maternity, leading or carrying forward their rosy boys and girls, for exhibition.

Some will smile, perhaps, in derision, at the idea of such an exhibition, but let me say that premiums given, for the finest children, (in the sense in which finest is here used) would induce many parents to investigate the laws of the animal economy, to learn what kinds of diet, how much exercise, and what character of exercise is necessary for a full development of man's physical power. Nay, more; mothers might thence learn, that health of children depends in a great degree upon the health of parents. Health of parents depends upon the observance of certain physiological laws, not generallly understood, and of course but little observed. These laws ought to be generally known; but how can they be known unless the attention of the people is particularly directed to them?

Pardon me, gentlemen, if you think I am dictating to your better judgment. I merely wish to call your attention to the subject of awarding premiums, for the finest children to be exhibited at our annual fairs, with a view to human improvement. I trust you will bestow that attention to the subject that its importance deserves.

---

Our Proverbs.—Listen if you would learn. Be silent if you would be safe. Inquire about your neighbor before you travel. The first of wisdom is the fear of God. The world is carrion, and its followers dogs. Poverty without debt is independence. Long experience makes large wit. The sluggard becomes a stranger to God, and an acquaintance with Indigence. By six qualities may a fool be known: Anger without cause, speed without profit, change without motive, inquiry without an object, putting trust in a stranger, and wanting capacity to discriminate between a friend and a foe.

